# Training in Rhythmic Gymnastics During the Pandemic

**DOI:** 10.3389/fpsyg.2021.658872

**Published:** 2021-04-09

**Authors:** Marta Bobo-Arce, Elena Sierra-Palmeiro, María A. Fernández-Villarino, Hardy Fink

**Affiliations:** ^1^Physical Education Department, Faculty of Sports Sciences and Physical Education, University of A Coruña, A Coruña, Spain; ^2^Especial Teaching Department, Faculty of Education and Sports Sciences, University of Vigo, Vigo, Spain; ^3^International Gymnastics Federation, Education Commission, Lausanne, Switzerland

**Keywords:** COVID-19, confinement, coaches, training, gymnasts, rhythmic gymnastics

## Abstract

The pandemic caused by the COVID 19 Virus creates an unprecedented situation of global confinement altering the development of competition and sports training at all levels of participation and in all sports, including rhythmic gymnastics (RG). To avoid possible effects of physical, technical and psychological detraining, coaches looked for home training alternatives. The objectives of the study were to know how rhythmic gymnastics training developed during the lockdown period (the conditions, type of training, performance monitoring means, and determinants of gymnasts’ participation) and to provide recommendations for a possible future lockdown. Three hundred and two RG coaches from twenty-six different countries throughout the five continents and four professional levels took part in the study: national team (28), international (26), national (172) and regional (75). The data collection tool was a questionnaire consisting of 39 closed questions structured in three dimensions: identification data of the coaches, training data during confinement and gymnast participation data. The independent variable was the gymnasts’ performance levels and the dependent variables organized in four categories: the technical media used to conduct and monitor the training sessions, the type of training done, the mechanisms for monitoring training performance and the aspects that determined participation. Most coaches kept their gymnasts training during confinement, although 76.5% confirm abandonment of any of their gymnasts. The main means used were real-time video conferencing, although at the lower practice levels the media stand out in deferred time. The contents of the training were mainly body technique, physical preparation and body difficulties. For performance monitoring, challenges, physical, and technical tests were predominant. The determinants for the development of training in the confinement vary depending on the level of the gymnasts, connectivity and electronic resources at the highest level, and the availability of spaces and social distancing at lower levels. For future lockdowns, it is necessary to review the content of the trainings, as well as the performance evaluation and the means necessary for it.

## Introduction

In December 2019, a severe acute respiratory syndrome outbreak caused by the Coronavirus 2 (SARSCoV-2 or COVID-19) occurred. The virus spread rapidly across the globe causing a pandemic without precedent and forcing governments to impose a global lockdown, giving rise to an extreme situation never seen before.

The world of sport, and of course also rhythmic gymnastics (RG), has been affected by the effects of pandemic and confinement in an unprecedented way, through the suspension of all kinds of sporting events, such as national competitions, international tournaments and macro events like the Olympic Games (JJOO). For its part, the situation of isolation has resulted in a number of direct consequences for the practice of sport: absence of organized training and competition, lack of physical communication between athletes and coaches, inability to move freely, lack of adequate exposure to sunlight, and inadequate training conditions (Jukic et al., 2020). Furthermore, Edwards and Thornton (2020) point out that this pandemic scenario may have triggered or worsened pre-existing psychiatric illnesses, such as anxiety, obsessive compulsive disorder (OCD), depression, insomnia and isolation. To this situation of affecting the well-being and self-esteem of athletes, coaches have had to face dilemmas such as controlling the performance of athletes, injuries and other information related to training (Evans et al., 2020). All this has limited the effectiveness of training methods, especially in sports of high technical and physical component, as is the case of RG.

RG is a sports specialty of great technical demand and high number of difficulties of extreme coordination and aesthetic complexity (Vernetta et al., 2017). Competition exercises involve mastery of five manual apparatus (rope, hoop, ball, clubs, and ribbon) in combination with body elements involving various components for high performance: physical, technical, tactical and psychological factors (Douda et al., 2007; Di Cagno et al., 2009). Practiced at individual or group (five gymnasts) modality, it is an artistic and aesthetic sport performed to the music with a particular training process: very young athletes, early specialization, high volume of training, high number of repetitions and great psychological stress (Bobo-Arce and Méndez-Rial, 2013; Debien et al., 2019).

We can distinguish different levels of competition (Rodríguez and Gómez-Landero, 2017) depending on the ages and the level of performance, from the highest (national team), to the lowest (regional level). But all require a specific training ranging from the work of body and apparatus technique, ballet or the integration of physical preparation and specific psychological preparation of sport (Law et al., 2007; Batista et al., 2018).

During the lockdown, training had to adapt to this unique situation in order to maintain the physical and technical condition of athletes. Given that physiological adaptation is a reversible process and that most of the aspects that determine it are lost during an extended period of inactivity, it is likely that during confinement there could have been a loss of up to 10% fitness for each week of total inactivity (Varandas et al., 2017; Eirale et al., 2020). Authors, such as Jukic et al. (2020) refer to changes after 8 weeks of detraining in flexibility, a fundamental quality in RG. In this same line, lack of training can also lead to the onset or worsening of pathologies, as well as a weight gain (Eirale et al., 2020; Sarto et al., 2020). Other authors mention a return to initial values in some capacities such as aerobic performance or strength in a 2 to 4-week period (Mujika and Padilla, 2001; Sousa et al., 2019). Tran et al. (2017) found a reduction in the sensorimotor capacity of athletes, which could be an important consideration when technical and/or skillful actions are required, as in the case of gymnastics. For their part, Edwards and Thornton (2020) point out that it is common for athletes to encounter transitional phases during their careers, although always foreseeable and under control. However, when the interruption is against their will, the consequences are comparable to those associated with an injury or can bring forward retirement from sport altogether, forcing the sportsperson to embark on a new path they had never planned for.

There are several studies on the impact of COVID-19 on athletes (Toresdahl and Asif, 2020) or on the conditions necessary for the resumption of training (Herrero-Gonzalez et al., 2020). However, the impact that confinement has had on training has received little attention and few research describes what has been done during this stage at home (Eirale et al., 2020; Herrera-Valenzuela et al., 2020; Jukic et al., 2020; Latella and Haff, 2020). In particular, none found in RG or similar artistic sports.

The concept of detraining as a total or partial loss of training adaptations and their application to a confinement situation and their prevention are totally novel phenomena (Girardi et al., 2020). Since health authorities have warned of future waves of COVID-19 and the possibility of encountering similar scenarios, it seems appropriate to know the conditions and difficulties in which rhythmic gymnastics training has developed during the confinement period. In this way, similar future situations can be oriented and planned, trying to minimize the effects that may have occurred on the loss of adaptation on gymnasts.

Therefore, the study aims to know how rhythmic gymnastics training has developed during the lockdown period (the conditions, type of training, performance monitoring means, and determinants of gymnasts’ participation) and to provide recommendations for a possible future lockdown.

## Materials and Methods

### Data Collection Tool

The data collection instrument was a questionnaire drawn up specifically for the occasion. It consists of 39 closed questions, structured in three dimensions. (i) Identifying data of the coach: country, federation, professional level, years of coaching experience and level of gymnasts they train. (ii) Training data during confinement: sports period in which confinement occurs, duration of the same, means or instruments used to direct training, training content, volume (days and hours per week), mechanisms of control and monitoring of gymnasts. (iii) Participation of gymnasts: monitoring of trainings, aspects that conditioned participation.

Discussion groups were set up with rhythmic gymnastics methodological and training experts to draw up the questionnaire and oversee its validation process. The profile of the experts corresponded to university professors with more than 20 years of experience in sports performance and to members of the International Gymnastics Federation’s (FIG) Scientific and Academic Commission, which is in charge of official gymnastics training and development programs at global level.

In the process of preparing the questionnaire, there were consultation rounds among the experts to obtain a consensual thinking of the group and to make a first draft of the questionnaire (Reguant-Álvarez and Torrado-Fonseca, 2016). From here, the discrepancies between the experts went through analysis and discussion, and a final report was prepared to achieve stability and consensus among the views of the group. In this way, the drafting process completed with the writing of the final questionnaire.

To ensure methodological and content validity, the questionnaire was resubmitted to expert judgment that gave their report on it and advised on possible modifications. The final version was the questionnaire presented below in the format used (online) and in three languages:

Spanish: https://docs.google.com/forms/d/1Qe2-kp4uc2-2ug8WO3LB0shaKVtgcDoXA7BsX5sR_OE/edit?usp=sharing

English: https://docs.google.com/forms/d/1oOhN_mNkG-jHddTIaVuDChb0334CGj0eWIVmxytF80/edit?usp=sharing

Russian: https://docs.google.com/forms/d/1JJO8GzrJwHn76jVdI82npOk5DDteViIEMvLrJMF4oJU/edit?usp=sharing

The internal consistency index for questionnaire questions was Cronbach Alpha coefficient obtaining values of α > 0.65.

### Procedure

Distribution was via email sending it to 150 FIG-affiliated Gymnastics Federations and Unions. Prior to the completion of the questionnaire, all participants received information of the purpose of the study, the anonymity of the answers and the processing of the data. They accessed it after giving their informed consent. Data collection lasted from July to November 2020, being that the time allowed for the completion of the questionnaire. The study developed in line with the ethical patterns of Sports Sciences (Harriss and Atkinson, 2015) and overseen by the Director of the FIG Academy.

### Sample

The sample comprised 302 RG coaches (age: 35.7 ± 121 years), from 26 countries across the five continents. Their professional level was national team 5%, international 16.6%, national 55.6%, and regional 22.8%. Considering the sports level of the gymnasts they coach, national teams 8.6%, international 8.6%, national 56.63%, and regional 26.5%. Most of the coaches in the study have more than 10 years of experience (59.3%), 24.5% experience between 5 and 10 years and 16.2% have been training for less than 5 years.

### Variables and Statistical Analysis

The independent variable studied has been the level of sport performance of the gymnasts: national team, international, national and regional.

The dependent variables studied have been set into four categories. Technical means used to direct and control trainings: real-time, deferred, others. Type of training carried out: physical preparation, ballet, body technical work, apparatus technical work, body difficulties, apparatus difficulties, parts of the competition exercise, psychological preparation and others. Training control mechanisms: weight, diet, physical tests, technical tests, challenges, others; Determining aspects of participation: connectivity, spaces and material, overload and injury, demotion and distance, loss of targets, others.

Within each dependent category, the variable others was included to give the coaches the opportunity to mark other possible less common options considered in their trainings, even though the specific content was not collected since it was a closed questionnaire distributed worldwide.

Statistical analysis was performed using SPSS version 25.0 for Windows (SPSS Inc., IBM, Armonk, NY, United States). Descriptive statistics used for variable description and study contextualization: means, standard deviations, frequencies, and percentages. The chi-squared test was used to identify the differences between gymnast performance levels and Cramer V to assess the degree of association between variables. The level of statistical significance was set at *p* < 0.05.

## Results

The results show that the average number of days of confinement was 107.5 ± 49.4, more than 3 months. During this time, gymnasts trained at home an average of 3.8 ± 1.3 days a week and 2.6 ± 1.3 hours per day. For most gymnasts, the confinement period coincided with the competitive period (70.9%) or pre-competitive (21.2%). Most coaches continued to do their training during the confinement period with more than 50% of their gymnasts (see Table 1), although a large majority, 76.5% refer to abandonment of some gymnasts during confinement.

**TABLE 1:** General characteristics of training during confinement.

Training volume	Mean	SD
Days of confinement	107.5	± 49.4
Training days per week	3.8	±1.3
Training hours per day	2.6	± 1.3
**Training period in which lock down happened**	**N**	**%**
Competitive	214	70.9
Pre-competitive	64	21.2
Pre-season	11	3.6
Resting	2	0.7
**Gymnasts following training**		
Less than 50%	67	22.2
More than 50%	175	56.6
All	64	21.2
**Any gymnasts leaving the sport?**		
Yes	223	76.5
No	71	23.5

**SD, standard deviation; N, frequency of answers.**

Results on the percentages of gymnast dropping out the sport during confinement (see Table 2) indicate that increased significantly in the groups of lower-level gymnasts (regional and national).

**TABLE 2:** Coaches answers on gymnasts dropping out the sport during confinement.

	Regional *n* = 75		National *n* = 172		International *n* = 26		Nat. Team *n* = 28		χ^2^	p	V Cramer
	N	%	N	%	N	%	N	%			
Yes	79	79.0	155	81.2	10	37.0	10	33.3	49.918	0.000	0.379
No	21	21.0	36	18.8	17	63.0	20	6.7			

**N, frequency of answers; n, sample size.**

The media used to conduct and monitor training sessions during the confinement period are in Table 3.

**TABLE 3:** Media used during training.

	Regional *n* = 75		National *n* = 172		International *n* = 26		Nat. Team *n* = 28		χ^2^	p	V Cramer
	N	%	N	%	N	%	N	%			
In real time	75	100.0	172	100.0	26	100.0	28	100.0	–	–	–
Deferred	52	69.3	118	68.6	12	46.2	14	50.0	8.441	0.038	0.167
Others	6	8.0	28	16.3	9	34.6	10	35,7	16.493	0.001	0.234

**N, frequency of answers; n, sample size.**

Regardless of the gymnasts’ levels, all the coaches used real-time video conferencing to enable themselves to conduct training sessions. Video, email and phone calls were used as complementary resources to the former, although in the case of the lower-level gymnasts the latter played a significant role (χ^2^ = 8,441; *p* < 0.05; moderate).

The different training content developed during the confinement period is shown in Table 4. Body technique was the most frequently used content during all of the training sessions, with no significant differences found in terms of the gymnasts’ performance levels (χ^2^ = 1.758; *p* > 0.05). Physical fitness and body difficulties were the other two content elements also common to all of the gymnasts, although they played a more significant role the higher the performance level. In the case of the international and national-team gymnasts, both ballet and technical apparatus work featured prominently in training sessions. Performing parts of competition exercises was the content element least developed by the gymnasts. Psychological training also represented a small percentage of the overall training content, increasing significantly the higher the gymnasts’ level (χ^2^ = 15.127; *p* > 0.02).

**TABLE 4:** Type of training done.

	Regional *n* = 75		National *n* = 172		International *n* = 26		Nat. Team *n* = 28		χ^2^	p	V Cramer
	N	%	N	%	N	%	N	%			
Physical	64	85.3	166	95.5	26	100.0	26	92.9	12.955	0.005	0.207
Ballet	36	48.0	123	71.5	24	92.3	27	96.4	32.705	0.000	0.330
Body tech*	73	97.3	16	95.3	25	96.2	28	100.0	1.758	0.624	0.076
Apparatus tech*	43	57.3	133	77.3	22	84.6	27	96.4	20.971	0.000	0.264
Body diff**	61	81.3	165	95.9	24	92.3	26	92.9	14.687	0.002	0.221
Apparatus diff**	32	42.7	118	68.6	20	76.9	14	50.0	18.993	0.000	0.251
Parts exercise	25	33.3	85	49.7	13	50.0	7	25.0	10.187	0.017	0.184
Psychological	24	32.0	83	48.3	19	73.1	16	57.1	15.127	0.002	0.224
Others	19	25.3	48	27.9	12	46.2	7	25.0	4.536	0.209	0.123

N, frequency of answers; n, sample size; (* technique; ** difficulties).

Table 5 shows the main evaluation and performance control means used by the coaches. Challenges come above all the others, followed by the technical and physical tests. In the case of the latter, the technical tests were carried out to a largely by the international gymnasts and the national team gymnasts. Weight featured more prominently as the gymnasts level of performance increased, and statistically significant differences were found in this respect (χ^2^ = 26.465; *p* < 0.05; moderate).

**TABLE 5:** Evaluation and monitoring means.

	Regional *n* = 75		National *n* = 172		International *n* = 26		Nat. Team *n* = 28		χ^2^	p	V Cramer
	N	%	N	%	N	%	N	%			
Weight	2	2.7	19	11.8	8	32.0	9	34.6	26.465	0.000	0.300
Food intake diet	6	8.0	32	18.9	6	25.0	3	11.5	6.490	0.090	0.149
Physical tests	32	42.7	57	33.3	14	56.0	13	48.1	6.723	0.081	0.150
Technical tests	29	38.7	72	41.9	14	53.8	13	46.4	2.026	0.577	0.082
Challenges	59	78.7	143	83.1	21	80.8	22	78.6	0.868	0.833	0.054
Others	12	16.0	37	21.5	8	30.8	17	60.7	24.107	0.000	0.283

**N, frequency of answers; n, sample size.**

The aspects that determined training development during confinement varied significantly in terms of the gymnasts’ levels (see Table 6). In the case of the international gymnasts and national teams electronic resources and connectivity were key, while for the gymnasts at regional and national levels availability of spaces and materials as well as the lack of motivation caused by social distancing from their teammates were fundamental.

**TABLE 6:** Determinants of gymnasts’ participation.

	Regional *n* = 75		National *n* = 172		International *n* = 26		Nat. Team *n* = 28		χ^2^	p	V Cramer
	N	%	N	%	N	%	N	%			
Electronic sources/connectivity	46	61.3	107	63.3	21	80.8	22	84.6	7.832	0.050	0.173
Spaces and materials	61	81.3	143	83.1	120	76.9	13	46.4	19.802	0.000	0.256
Overload and injuries	12	16.0	41	23.8	10	38.5	8	28.6	5.979	0.113	0.141
Demotivation and distancing	65	86.7	140	81.9	15	60.0	10	35.7	37.189	0.000	0.353
Loss of sports goals	54	72.0	143	83.1	17	65.4	19	67.9	8.095	0.044	0.164
Others	24	32.0	49	28.5	8	30.8	12	42.9	2.386	0.496	0.089

**N, frequency of answers; n, sample size.**

It is notable that during this period, overloads and injuries did not play a decisive role in the participation of any of the gymnasts.

## Discussion

The aims of this study were to know how rhythmic gymnastics training developed during the lockdown period (the conditions, type of training, performance monitoring means, and determinants of gymnasts’ participation) and to provide recommendations in the event of a future lockdown. Most of the scientific literature on COVID-19 in sport tackled the direct impact of the pandemic on social, health and organizational aspects or on strategies for the return to training and competition. However, the training done in confinement, what the conditions were and what the consequences of this period were with regard to rhythmic gymnastics have scarcely been looked at.

The results indicate that for most gymnasts the confinement period coincided with the time of the main competitions, which logically could not take place. Most continued to train with a high volume of training in both days and hours, which could be due to the high levels of both coaches and gymnasts. The majority belong to national teams with fundamentally international participation at the highest level, world championships and Olympic Games. The results described by Bowes et al. (2020) on the impact of the pandemic on elite women’s sport go along these lines. They point out that 100% of those surveyed continued doing their sporting activity and received the support of the coaching staff via online training sessions, this being the tool most used by coaches with their gymnasts in our study.

In relation to the duration of training sessions, Eirale et al. (2020) recommend no more than 60 min per day, both for developing strength and endurance. Ranasinghe et al. (2020) indicate that after an hour, during periods of vigorous training or competition, there is a reduction in the number of immune cells circulating in peripheral blood, therefore they recommend having gradual training strategies with no excessive doses during and after confinement. The rhythmic gymnasts in our study amply exceed the average of this number of minutes.

Of the few studies on training content, Jukic et al. (2020) stress the difficulty involved in monitoring the training load, especially with regard to intensity and volume, as well as prescribing the type of exercise precisely. The RG coaches in our study were capable of developing specific exercises such as ballet or body and apparatus technique, whose loads were difficult to monitor, as they mainly oversaw weight and diet. Eirale et al. (2020) emphasize that excessively intense training can weaken the immune system, thus increasing the risk of catching COVID-19. Hence, the scant use of monitoring mechanisms in the sample may give rise to this undesired situation. Hagen et al. (2020) recommend using welfare questionnaires and remote monitoring tools (mobile apps, GPS, temperature, heart rate monitoring, etc.) to evaluate the state of physical performance and strategically plan for their return to sporting activity when training, as well as competition, is resumed under normal conditions.

Physical fitness was the content most used with the rhythmic gymnasts in the study. Several authors point out that during confinement they worked on strength, overall fitness, endurance training and sports skills (Jukic et al., 2020; Latella and Haff, 2020; Melim et al., 2020). On the other hand, Herrera-Valenzuela et al. (2020) in a study carried out on combat athletes, recommend high-intensity interval training (HIIT) and sport-specific muscular strengthening. Along these lines, Melim et al. (2020) describe HIIT as the most suitable for footballers. Also worthy of highlighting is the importance the RG coaches in the sample place on body and apparatus technique skills, coinciding with the recommendations made by several authors with regard to coaching sport-specific technical skills (Jukic et al., 2020; Ranasinghe et al., 2020). However, other key technical contents of the sport, like apparatus difficulties (many of them performed to high throws into the air of the apparatus) and parts of the competitive exercise (complex connections of body and apparatus movements done at a floor area of 13 × 13 m) were scarcely included.

The psychological preparation of the gymnasts in the study was a training content with little consideration at all levels of performance, but the national teams, when the majority of authors refer to the lockdown as an ideal moment for this kind of training (Bowes et al., 2020; Latella and Haff, 2020; Ranasinghe et al., 2020). Studies in the prolonged abstinence from physical training and its psychological effects in athletes, forced to abstain due to injury, show that they are more likely to develop problems such as depression, anxiety, loss of self-esteem and mood swings (Melim et al., 2020). At different levels of performance and before the times of COVID pandemic, the RG psychological studies dealt mostly with anxiety, attentional and pre-attentional processes, self-consciousness, behavior analysis and personality traits of rhythmic gymnast (Bobo-Arce and Méndez-Rial, 2013). Cénat et al. (2021) in a systematic review of the psychological effects of the pandemic, with more than 190,000 participants, found that depression, anxiety and stress were between five and three times more frequent than commonly reported by the World Health Organization (2017). Effects that have not been analyzed yet in RG gymnasts across the world and at different levels of performance. Future studies should approach the consequences of the global lockdown to determine the specific effects on gymnasts and to explore if the sport practiced contributed to reduce or increase them.

A study carried out with Italian athletes (Di Fronso et al., 2020) found a significant increase in perceived stress and dysfunctional psycho-biosocial states in comparison with pre-confinement situations, greater in women than in men, and greater in novice than in elite athletes. Esteves et al. (2020) describe similar results for professional footballers, pointing out that high performance male athletes have lower levels of anxiety. It appears to be desirable, to include in gymnasts’ training sessions specific psychological programs directed to female athletes at non-professional level, as recommended by the UN in order to prevent the pandemic from influencing gender and social inequality (United Nations, 2020). Iancheva et al. (2020) find also differences between men and women in sports specialties and refer to differences in psychological intervention. Furthermore, these authors found different ways of perceiving the consequences of the pandemic in accordance with nationality, which makes it easier to understand some of the results obtained in this study.

Even though psychological training has not been a priority for the coaches during this first world lockdown, it is remarkable that challenges were the main monitoring mechanism used. Considering that challenges could represent a motivation for gymnast to keep on training gymnastics skills under difficult circumstances, it is to some extent contradictory the results obtained in the two categories: training content and monitoring means.

With regard to the difficulties sportspeople found in continuing their training routines during the confinement, Bowes et al. (2020) point out that 94% told of problems that affected their training regimes, and 74% of them indicated mainly that they were unable to train in suitable, specialist facilities, or use specialist equipment. A high percentage of coaches in this study indicate that they had some gymnast who left due to the difficult circumstances of confinement. The results also show significant differences according to the levels of performance. For the elite gymnasts extrinsic factors, such as poor connectivity or lack of facilities and materials are the main reasons behind problems in training. For the lower level gymnasts, intrinsic factors including the lack of contact with other gymnasts and the loss of sport goals were determinant.

It is notable that for all performance levels in the study overloads and injuries did not play a decisive role, even though the difficulty of implementing an injury recovery plan with the scant resources available for professional and physical assistance in the home is considered as a limitation (Girardi et al., 2020).

There is no data found in previous studies on athletes abandoning training during the lockdown. Rhythmic Gymnastics is taken up from a very early age, therefore gymnasts will be psychologically immature and their predisposition to doing sport maybe modified by such a long period of confinement. For youth sports, Breslin et al. (2020) recommend guidance and working in three priority areas: mental health and dealing with uncertainty, maintaining social connections, and motivation and setting goals. The results also indicate that giving up is more frequent the lower a gymnast’s level, which might suggest that being less involved in sport can lead to more people giving it up altogether. Similarly, the type of rhythmic gymnastics most participants do at lower performance levels is in groups (five gymnasts together), and this was unfeasible during confinement. While extrinsic conditions for training in a lockdown situation could improve through sport policies and investment, intrinsic factors should be deal through specific psychological training of the gymnasts.

Bearing in mind the study’s limitations (sample size, the distribution of training loads and the type of gymnastics modality, that is individual or group performance), and the scientific evidence with respect to training during the period of confinement, we would propose the following recommendations for RG in case of subsequent home confinements:

• To plan the training load (intensity and volume), considering the different content possible beyond fitness training and body and apparatus technique. It would be necessary to include other main components of the sport that the gymnasts can practice at home, like choreographic preparation based on ballet, expression and musical rhythm, and mental preparation, using concentration and visualization techniques.
• To establish the duration of training sessions at home in accordance with gymnasts’ ages and performance levels, avoiding prolonged sessions of over an hour when the training content is of high intensity.
• To program the calendar and mechanisms needed for monitoring gymnasts’ evolution in all dimensions of performance: physical, technical and psychological.
• To provide the gymnasts with the resources necessary for them to develop their training sessions and control their performance progressions at home.
• To draw up specific psychological support programs to give guidance and help gymnasts to maintain their motivation and goals in the sport.

## Conclusion

During the period of confinement brought about by the COVID-19 RG training was widely monitored by the coaches in the sample as well as by the gymnasts themselves, although a high percentage of coaches admit that some of their gymnasts gave up the sport altogether. The technological resources used enabled coaches and gymnasts to be in contact and to prescribe and follow training sessions online. This did not occur to the same extent in the monitoring of training, which was scant and somewhat vague.

The training content most used was general and sport-specific physical fitness as well as body and apparatus technique. The average duration of training sessions was much higher than that recommended in other studies on athletes’ health and well-being. For its part, psychological preparation was not undertaken enough during the lockdown period, contrary to the recommendations found in literature and which should be corrected in the event of future confinements by establishing specific protocols.

The difficulties revealed with regard to the continuation of training, such as connectivity problems and lack of facilities and materials, should be resolved at some stage in the future.

## Data Availability Statement

The raw data supporting the conclusions of this article will be made available by the authors, without undue reservation, to any qualified researcher.

## Ethics Statement

Written informed consent was obtained from the individual(s) for the publication of any potentially identifiable images or data included in this article.

## Author Contributions

MB-A and ES-P contributed to the study conception and design. MB-A and HF controlled the validation process and data collection. MF-V performed the data analysis. MB-A, ES-P, and MF-V wrote, reviewed, and edited the manuscript. All authors contributed to the article and approved the submitted version.

## Conflict of Interest

The authors declare that the research was conducted in the absence of any commercial or financial relationships that could be construed as a potential conflict of interest.
